# Possible Interaction of Suramin with Thalamic P2X Receptors and NLRP3 Inflammasome Activation Alleviates Reserpine-Induced Fibromyalgia-Like Symptoms

**DOI:** 10.1007/s11481-025-10207-4

**Published:** 2025-05-07

**Authors:** Maram M. Mohamed, Hala F. Zaki, Ahmed S. Kamel

**Affiliations:** Department of Pharmacology and Toxicology, Faculty of Pharmacy, Cairo University, Cairo, Egypt

**Keywords:** Thalamus, Fibromyalgia, Purinergic receptors, Inflammasome signaling, Neuroinflammation, Suramin

## Abstract

The high pain sensitivity in fibromyalgia (FM) is processed by the thalamus that presents as a key component in the pain pathway in FM patients. Noteworthy, Purinergic receptors, specifically P2X, are implicated in pain signaling and neuroinflammation via inflammasome signaling. However, there is no available data on the impact of pharmacological intervention on the P2X receptor in thalamic pain transmission in FM. To investigate this aspect, the clinically tested P2X inhibitor, Suramin (SURM), was utilized. FM was induced over three days using Reserpine (1 mg/kg/day, s.c.), followed by a single dose of SURM (100 mg/kg, i.p.). At the molecular level, SURM countered the overexpression of P2X7 and P2X4 receptors accompanied by reduced NLRP3 inflammasome complex and pyroptotic markers like gasdermin-D. This was associated with the suppression of the p38-MAPK and NF-κB pathways, along with a decrease in pro-inflammatory cytokines and tumor necrosis factor-α as observed by increased CD86 expression on M1 microglia phenotype, a neuroinflammatory marker. Concurrently, blocking the P2X receptor shifted microglia polarization towards the M2 phenotype, marked by elevated CD163 expression, as a neuroprotective mechanism. This was outlined by increased neurotrophic and anti-inflammatory IL-10 with normalization of disturbed neurotransmitters. Behaviorally, SURM ameliorated the heightened pain processing, as observed in mechanical and thermal pain tests. Furthermore, it lowered Reserpine-induced motor impairment in the rotarod and open-field tests. This improvement in the somatosensory experience was reflected in alleviating depressive-like behavior in the forced swimming test. These findings highlight the therapeutic potential of blocking thalamic P2X receptors in alleviating fibromyalgia symptoms.

**Graphical Abstract:**

Thalamic Purinergic Inhibition abrogates Fibromyalgia-associated pain

Blocking Thalamic P2X receptors by Suramin has therapeutic potential of alleviating pain and neuroinflammation associated with fibromyalgia symptoms**.**

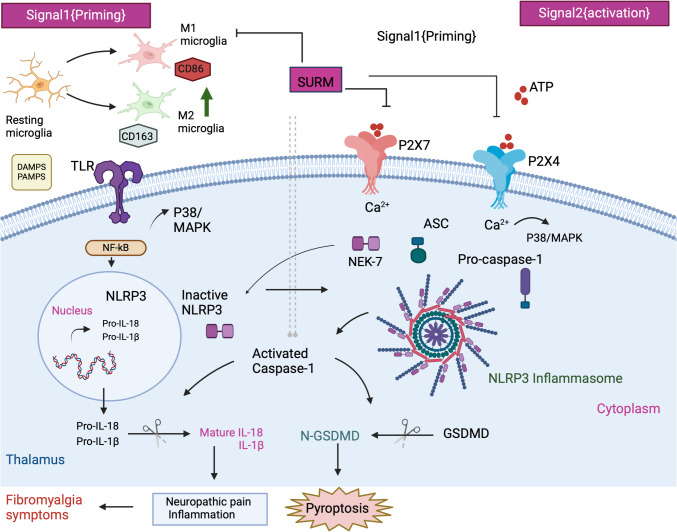

**Supplementary Information:**

The online version contains supplementary material available at 10.1007/s11481-025-10207-4.

## Introduction

Fibromyalgia (FM) is a chronic condition characterized by widespread musculoskeletal pain. This syndrome is often accompanied by coordination difficulties and persistent fatigue. Also, FM comorbidities exacerbate the primary symptom of pain (Wolfe et al. 2010). The FM typically presents in middle-aged females compared to males (3:1) (Queiroz 2013). Notably, the thalamus in spinothalamic tract is a relay station for pain transmission in the nociceptive pathway. These neuronally-driven responses contribute to the formation of central sensitization in chronic pain situations. In an early study, functional magnetic resonance imaging (fMRI) revealed that FM people had an imbalance in thalamus connectivity (Cifre et al. 2012). In addition, women who have fibromyalgia showed altered regional cerebral blood flow (rCBF) in the thalamus (Mountz et al. 1995; Adigüzel et al. 2004; Foerster et al. 2011). Notably, Kim et al. 2021 found reduced connectivity between the left posterior thalamus and the inferior parietal lobule, which correlated with lower pain thresholds in FM patients compared to healthy controls (Kim et al. 2021).

The interplay between purinergic signaling and neuroinflammation with pyroptosis in central sensitization represents a promising area of investigation. This is marked by heightened pain response after purinergic P2X receptors activation in mouse thalamus subjected to hemorrhage-induced post-stroke pain (Huang et al. 2022). ATP is released under cellular stress or damage and over-activates the P2X receptor. This activation stimulates microglia with inflammatory cascade and pyroptosis, thereby perpetuating the cycle of pain and inflammation in FM (Giorgi et al. 2022). Research has highlighted the involvement of different purinergic receptors, particularly P2X4 and P2X7, in central sensitization and various pain states. The P2X4 receptor, for instance, is linked to the amplification of pain signals via microglia in the spinal cord, leading to allodynia (Inoue 2007). Moreover, the activation of P2X7 is known to provoke neuropathic pain in chronic inflammatory pain models (Itoh et al. 2011). This has been associated with the NLRP3 inflammasome pathway, triggering an inflammatory response in FM (D’Amico et al. 2021). Furthermore, activation of NLRP3 leads to the release of IL- 1β and IL- 18 that exacerbate painful sensations in neuropathic pain (Coll et al. 2022). All these show the necessity of therapeutic interventions targeting this signaling pathway to offer a novel approach to managing FM symptoms.

The present study evaluated the anti-inflammatory and anti-nociceptive effects of purinergic receptor inhibition on pyroptosis and microglial activation in the thalamus of the FM rat model. The study utilized a reserpine-induced FM model (RIFM) to fulfill this aim. It displays chronic muscle fatigue, monoamine content in the CNS and depressive-like behaviors (Arora and Chopra 2013), demonstrating the validity of using this model. A biogenic amine depletory agent, reserpine (RES), functions in the descending pain inhibitory system, where monoamines are the primary transmitters. When RES depletes monoamines, descending spinal nociceptive neurons exhibit an augmented response to noxious stimulation, leading to the manifestation of pain-centered symptoms of FM (Wood et al. 2007). Meanwhile, deficits in the thalamic DA and 5-HT are considered as hallmarks in the reserpine FM model (Nagakura et al. 2009).

To test the crucial role of purinergic receptors in thalamic pain signaling, the study utilized Suramin (SURM) as a purinergic receptor inhibitor. The antioxidant and anti-inflammatory properties are among the many potential therapeutic effects of SURM (Hawking 1978; Voogd et al. 1993). It significantly modulates the inflammatory response in various disease models via the NLRP3/caspase- 1/GSDMD pathway (Zhu et al. 2024). This suggested that SURM could be beneficial in alleviating symptoms of FM by targeting critical pathways involved in pain and inflammation.

## Materials and Methods

### Animals

From the National Research Center in Giza, Egypt, female Wistar albino rats weighing 180–220 g were obtained. One week before the experiment was conducted, the rats were acclimated to standard housing conditions at the Faculty of Pharmacy's Animal Facility in Cairo, Egypt. Moreover, water and a chow diet were provided ad libitum. The adaptation and the investigational periods were under controlled environmental circumstances (25 ± 2 °C; temperature, 60 ± 10%; humidity and 12/12 h light/dark cycle).

### Induction of the FM-Like Model

RES (Sigma-Aldrich, Saint Louis, MO, USA) was prepared in 0.5% glacial acetic acid solution. RES was administered subcutaneously in a dose of 1 mg/kg/day in the neck region for three days.

### Experimental Design

**Scheme 1:**
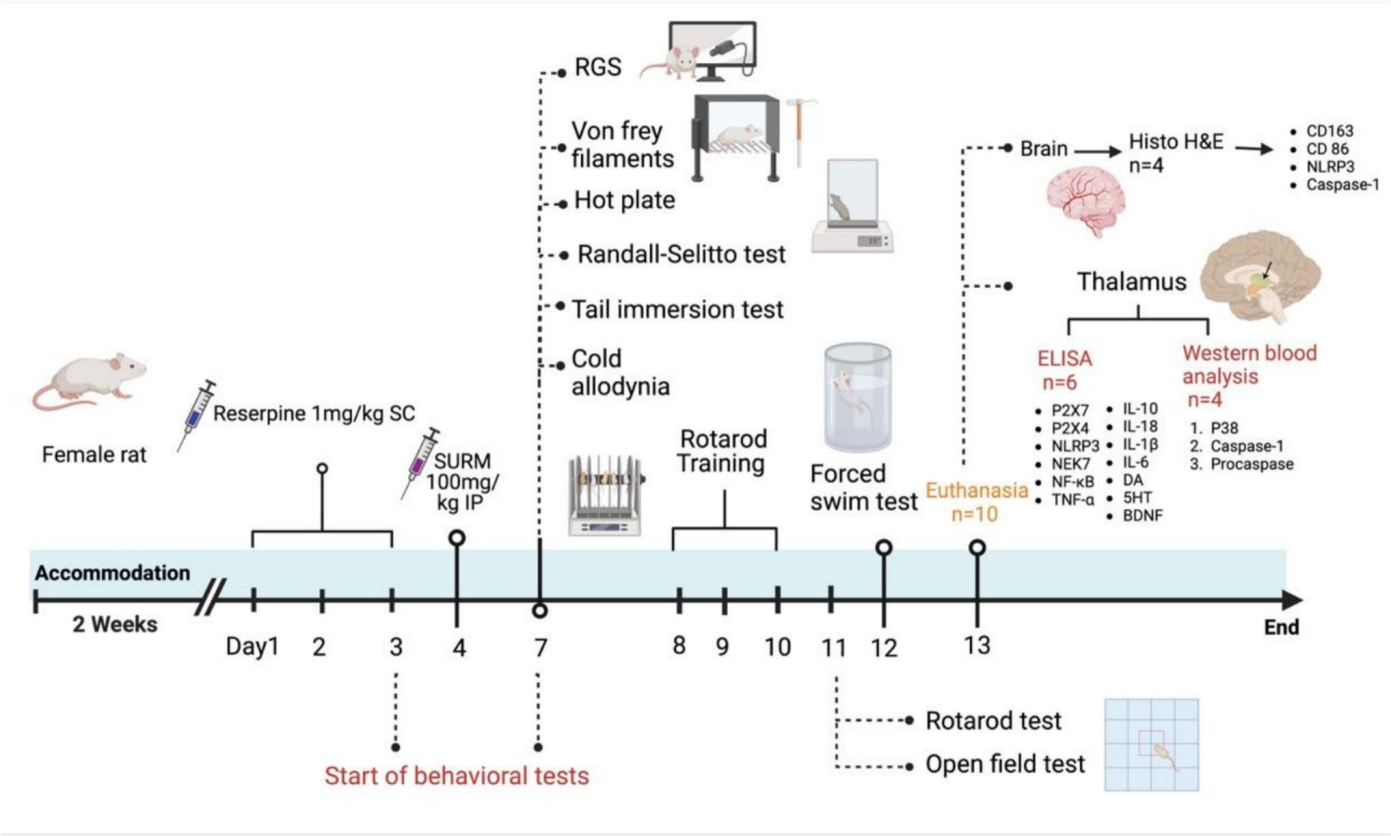
Experimental design representation of timeline for drug administration and behavioral tests

Thirty female rats were randomly assigned to three groups (n = 10/group) using a random number generator to eliminate allocation bias. The study utilized the G-power calculator version 3.1 (Düsseldorf, Germany) to validate the sample size. The parameters for the power analysis were: an effect size of 0.6, an alpha level of 0.05, and a power of 0.8. The first group was designated the control group and administered a 0.5% acetic acid solution as a vehicle. Group two was administered reserpine at a dose of 1 mg/kg subcutaneously to induce a model similar to FM. Group three was administered reserpine at a dosage of 1 mg/kg subcutaneously for three consecutive days, followed by a single intraperitoneal dose of SURM (Sigma-Aldrich, MO, USA) at a dosage of 100 mg/kg dissolved in sterile water for injection (Kharlamov et al. 2002; Naviaux et al. 2014). The study initially assessed spontaneous pain-related behaviors using the Rat Grimace Scale (RGS) before introducing any stimulus-evoked pain tests. Pain-like behaviors were then evaluated using Von Frey filament, Randall-Selitto test, cold allodynia, hot plate, and the tail immersion tests before and after SURM administration on days 3 and 7, respectively. On day 11, motor abnormalities were evaluated by open-field and rotarod tests. On day 12, depressive-like behavior was measured by a forced swimming test. These tests were conducted in order of least to most stressful, this order was chosen to prevent thermal stimulation from sensitizing animals to subsequent mechanical stimuli, which could lead to mechanical hyperalgesia (Gröne et al. 2012)**.** The tests were conducted in consecutive order, with a 2-h break between each test. Following the conclusion of the behavioral assessment, rats were euthanized by decapitation under the influence of a mild anesthetic consisting of ketamine (50 mg/kg) and xylazine (10 mg/kg) (Khajuria et al. 2012). The initial subset (n = 4) was prepared for histopathological and immunohistochemical examinations by fixing brains and spinal cords in 10% buffered formol saline. For subsequent analysis of the biomarkers using western blot (n = 4) and ELISA (n = 6) techniques, the thalami of the second subset (n = 6) were dissected, flash-frozen in liquid nitrogen, and stored at − 80 °C. Throughout the samples'analysis, the samples'identities were unknown to all inspectors. A different researcher was responsible for coding and decoding the samples. Researchers were blinded to group assignments during behavioral assessments and data analysis to minimize observer bias. The experimental design was illustrated as shown in scheme 1.

### Behavioral Tests

#### Rat Grimace Scale

The Grimace scale is a facial expression grading system that has been used to quantify animals'spontaneous suffering. Rats'facial expressions changed significantly and permanently, according to three raters who were blind to the treatment the rats received. Each animal was kept in its own acrylic cage with woodchips for bedding, a 12-h light/dark cycle, a controlled temperature (23 ± 2 °C) and humidity (55 ± 10%), and unlimited access to water and normal laboratory chow. Every test was carried out between 8:00 and 20:00 h of daylight. Then the raters assigned ratings to four action units for every image: changes in the features of the ears and whiskers, nose/cheek flattening, and orbital tightening. In accordance with the rating methodology outlined by Sotocinal et al. (Sotocinal et al. 2011), each action unit was assigned a 3-point rating (0 being not present, 1 being moderately present, and 2 being plainly evident). For each of the four action units, the average ratings were calculated. Additionally, the rat Grimace scale (RGS) score for each image was determined by averaging the three raters. Lastly, each animal's RGS score was determined by averaging the RGS scores of each rat's ten photos(Tanei et al. 2020).

#### Von Frey Hair Test

Mechanical allodynia was evaluated by observing a notable decrease in withdrawal thresholds during the von Frey hair test. Rats were placed collectively on an elevated mesh platform with 1 cm^2^ holes within a transparent plastic enclosure, allowing them to acclimate to the testing environment for at least 15 min. Von Frey hairs (obtained from IITC Life Science, Inc.) with pre-calibrated bending forces (measured in grams) were used to deliver precise mechanical stimuli of varying strengths. The test began with the filament exerting the least force, targeting the plantar surface of the left hind paw from beneath the mesh. Each hair was applied to the paw to cause a slight bend and held for 2–5 s. An interval of 6 to 8 s was maintained between each of the five stimulations. A positive reaction was recorded when the paw was quickly withdrawn. The sensitivity threshold of the paw was defined as the lowest force needed to elicit a withdrawal reflex, determined by the filament that led to a rapid paw withdrawal in 3 out of 5 attempts. Any movement associated with walking was not considered a withdrawal response (Deuis et al. 2017).

#### Tail Immersion Test

The tail-immersion test simulates acute heat-induced pain, assessing spinal thermal sensitivity by immersing a portion of the experimental animal's tail in a hot water bath. Specifically, one-third of the tail was submerged in hot water at 55 °C. The time it took for the rat to retract its tail, with a cut-off time of 15 s, was closely observed and recorded as the tail withdrawal latency (Mokhemer et al. 2023).

#### Randall-Selitto Test

The Randall-Selitto test assesses the threshold response to mechanical pressure stimulation. It involves applying gradually increasing mechanical force using a Randall-Selitto algesimeter. Each rat was gently restrained during the test, with its left hind paw positioned under a pressure pad. The force applied to the paw, measured in grams, was incrementally raised until the rat withdrew its paw due to intolerance to the pressure. This point marks the Randall-Selitto mechanical threshold. The pressure was promptly released upon paw withdrawal, and the force required to elicit this response was recorded (Santos-Nogueira et al. 2012).

#### Hot Plate Test

The hot plate test is a methodology employed to evaluate the susceptibleness of rats to heat. Each rat was meticulously positioned on a heated surface for this experiment. Rats initially react to the distress of a thermal stimulus by lapping their feet when exposed to a heated surface. Subsequently, they exhibit pronounced evasive behaviors, such as leaping. The temperature of the heated plate apparatus was 55 ± 1 °C. The time required for the rat to demonstrate rear paw lapping or leaping to evade the distress caused by the heat was recorded as the hot plate withdrawal latency. A maximum time limit of 20 s was enforced to prevent animal tissue damage (Kamel et al. 2022).

#### Cold Allodynia Test

The cold allodynia test evaluates the sensitivity of rats to cold temperatures. In this test, each rat's hind paw is carefully dipped into a container filled with ice-cold water, kept at a temperature of 4 ± 1 °C. The time taken for the rat to withdraw its paw, known as cold allodynia paw withdrawal latency which was then measured. Each session involved testing only one hind paw at a time, with a maximum duration of 20 s for each immersion to safeguard the animal's well-being. For every rat, two separate measurements were taken for each hind paw, spaced five minutes apart. The cold allodynia paw withdrawal latency was then calculated as the average of these readings from both hind paws. An extended cold allodynia paw withdrawal latency was interpreted as an anti-allodynic effect, indicating reduced sensitivity to the cold, while a shorter paw withdrawal latency suggested more severe allodynia, indicating heightened sensitivity (Kamel et al. 2022).

#### Forced Swim Test

In the forced swim test, a rat is placed in a water-filled container where it initially attempts to escape but eventually adopts a state of immobility, indicating depressive-like behavior. The test duration is six minutes, consisting of a two-minute pretest and four-minute test periods. During the test, the rat was placed in the water-filled container with no means of escape, and the water temperature was maintained at 23 ± 2 °C. Immobility in the rodent is recognized when it assumes a bent forward position with no movement in its rear legs. Observers unaware of the drug treatment manually record the duration of immobility (Yankelevitch-Yahav et al. 2015; Kamel et al. 2024).

#### Open Field Test

The open field test is a widely used method for assessing locomotion behaviors. It uses an open square wooden box (80 × 80 × 40 cm) with red walls and a white floor, divided into 16 equal squares by black lines. During the test, a rat is placed in the center of the box's bottom surface, and its movements are tracked and recorded using ANY-Maze video tracking software (Stoelting Co, USA) for 6 min. The parameters evaluated include the time spent in different areas of the open field traveled distance, open field mean speed, open field time immobile, open field latency time, and the number of entries into the central zone. The illumination level is maintained at 150 lx throughout the task (Avila et al. 2010).

#### Rotarod Test

The rotarod test is utilized to evaluate motor coordination in rats. This involves a cylindrical rod measuring 3 cm in diameter and 120 cm in length, rotating consistently at 20 rpm. Rats must maintain their balance on the rotating rod instead of falling onto a platform beneath it. Prior to the main experimental procedures, each rat undergoes five trial runs. Only animals that successfully remain on the rod for 5 min are selected for the experiment. Following these trials, the probe test was conducted, during which the time it takes for the animal to fall off the rod (fall-off latency) was recorded, with a maximum time limit of 300 s (Avila et al. 2010; Ibrahim et al. 2024).

### Histopathological Studies

Brain tissue sections were preserved for 72 h in 10% neutral buffered formalin. Afterward, samples were immersed in paraplast tissue embedding media after being purified in xylene and processed in ethanol of varying grades. Sagittal brain tissue sections, 5 μm in thickness, were subjected to rotatory microtome at the mid-thalamic regions. The sections were then adhered to glass transparencies. As a standard histological examination staining procedure, tissue sections were stained with hematoxylin and eosin (H & E). Subsequently, novice histologists conducted blinded examinations of the sections. They were then evaluated blindly by a skilled histopathologist. According to Culling (2013), all standard procedures for processing samples were performed (C.F. Culling 2013).

### Immunohistochemical Analysis

Tissue sections, 4 μm thick and embedded in paraffin, were treated with 3% H_2_O_2_ for 20 min following the manufacturer's protocol. After washing, the cells were incubated overnight at 4 °C with the following antibodies: anti-CD163 (Cat # GTX35247, GeneTex, CA, USA); anti-CD86 (Cat # bs- 1035R, Bioss, USA); and anti-CASPASE- 1 antibody (14 F468) [NB100 - 56565—1:100 Novus bio Co.]. The tissues were then treated with diaminobenzidine (DAB) for 10 min after rinsing with PBS, followed by incubation with the secondary antibody HRP EnVision reagent (DAKO) for an additional 20 min. After washing with PBS, the tissues were coverslipped for microscopic examination, counterstained with hematoxylin, dehydrated, and clarified in xylene. The mean optical density of thalamic cellular immunohistochemical expression levels of NLRP3 and caspase- 1, as well as the mean count of CD163 +  +/CD86 +  + reactive microglial cells per field, were determined by scanning at least six representative non-overlapping fields per tissue section of each sample, following the methodology of Mohamed et al. (2023) (Mohamed et al. 2023).Tissue sections were analyzed using Leica application software with a full HD microscopic imaging system (Leica Microsystems GmbH, Germany) operated by an experienced histologist examiner.

### Western Blot Analysis of P38 MAPK, Procaspase- 1 and Cleaved Caspase- 1

Following rinsing in PBS, the thalami were lysed utilizing the Ready Prep™ protein extraction reagent manufactured by Bio-Rad Inc. in California, USA. Protein concentration was determined using the Bradford assay. Proteins were separated on 10% SDS-PAGE gels using a Mini-PROTEAN system (Bio-Rad) at 120 V for 90 min. Proteins were transferred onto nitrocellulose membranes using a wet transfer apparatus (Bio-Rad) at 100 V for 1 h. Membranes were blocked in 5% non-fat milk in TBST buffer for 1 h at room temperature. Primary antibodies against Pro-caspase- 1 (Cat # ab179515, Abcam,USA), caspase- 1 (Cat # PA5 - 86,692) and p38 MAPK (Cat # PA1 - 30391), both of which were provided by (Thermo scientific, Rockford, Illinois, USA), were diluted 1:1000 in blocking buffer and incubated with the membranes overnight at 4 °C. After washing, membranes were incubated with HRP-conjugated secondary antibodies (1:5000 dilution) for 1 h at room temperature. Membranes were washed three times with TBST buffer for 10 min each. Proteins were visualized using the ECL detection system (Thermo Fisher Scientific) and imaged using a ChemiDoc imaging system (Bio-Rad). Band intensities were quantified using Image Lab software (Bio-Rad) and normalized to β-actin protein. Protein marker was supplied by FastGene (NIPPON Genetics EUROPE GmbH) to highlight the molecular weight of the detected proteins.

### Enzyme-Linked Immunosorbent Assay (ELISA)

Using rat-specific ELISA kits, the following neurotransmitters in the thalamus were determined; dopamine (My BioSource Cat # MBS7214676, San Diego, CA, USA), serotonin (5HT) (My BioSource, Cat # MBS9362408, San Diego, CA, USA), NLRP3 (Cat # MBS2033695, San Diego, CA, USA), NF-κB (Cat # MBS2505513, San Diego, CA, USA), BDNF (Cat # MBS824814, San Diego, CA, USA), IL- 1β (Cat # MBS825017, San Diego, CA, USA), IL- 10 (Cat # MBS2707969, San Diego, CA, USA) and TNF-α (Cat # MBS9362408, San Diego, CA, USA). Abeexa Elisa kits were utilized for assessing P2X4 (Cat # abx537443, Cambridge, UK) andNEK7 (Cat # abx535012, Cambridge, UK). In addition, Life Span BioSciences ELISA kits were used for quantification of P2X7 (Cat # LS-F16541, WA, USA) and gasdermin D (GSDMD) (Cat # LS-F39621, WA, USA). Furthermore, IL- 18 (Elabscience, Cat # E-EL-R0567, Wuhan, China) and IL- 6 (RnDSystems, Cat # R6000B, Minneapolis, MN, USA) were determined referring to the relevant kit's instructions. The results were presented as pg/mg protein for TNF-α, NF-κB, IL- 6, IL- 10, IL- 18, IL- 1β, and BDNF and as ng/mg protein for NLRP3, NEK7, GSDMD, P2X7, P2X4, DA, and 5HT. The total protein concentrations in tissue samples were measured by the BCA kit supplied by G-Bioscience (USA). To determine the level of the protein of interest, the study utilized the kit’s standard curve to calculate concentrations in pg/ml or ng/ml, which were then normalized by dividing by the tissue protein concentration (in mg/ml), yielding results expressed as pg/mg protein or ng/mg protein.

## Statistical Analysis

The data were presented as the mean ± standard deviation. For multiple comparisons, a one-way analysis of variance (ANOVA) was employed, followed by the Tukey post hoc test. The p-value threshold for significance was set at less than 0.05. GraphPad Prism 9.2.0 (San Diego, CA, USA) was used to perform statistical analysis. The terms"fold"and"%"in the Results section represent normalized values, calculated relative to either the control or model group. Comparisons of pain-like behaviors between groups were conducted using two-way repeated measures ANOVA followed by Tukey’s post hoc test to analyze pairwise differences between groups at baseline (day 3, pre-treatment) and post-treatment (day 7). The F-value (F), Eta squared (η2) and statistical significance (*p*) were calculated for each effect.

## Results

### Suramin Ameliorated Pain Anomalies and Elevated Mood in the FM-Like Model

**Fig. 1:**
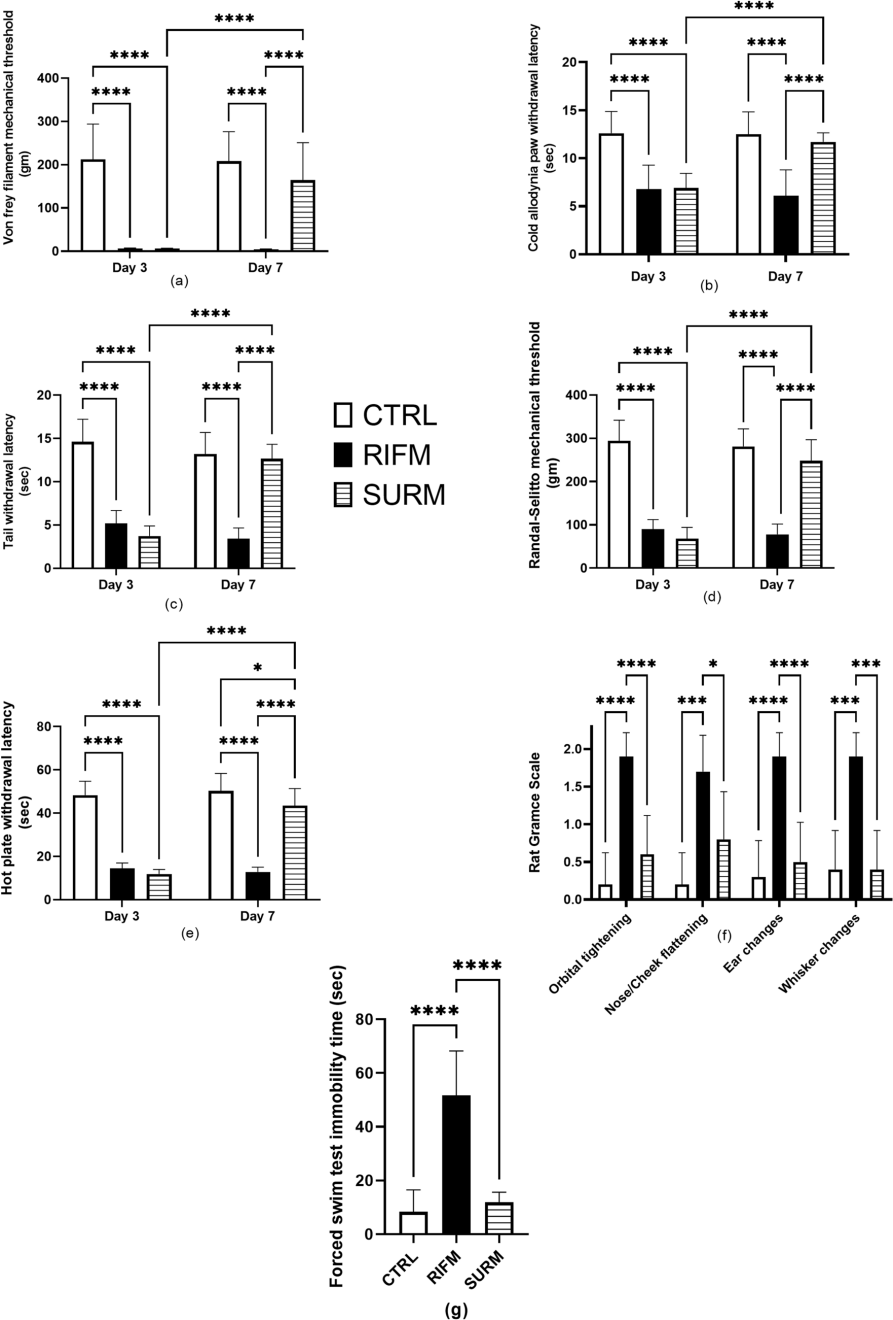
Effect of SURM on sensory impairments and depressive-like effect of RIFM rats. Panels represent: (a) mechanical threshold in Von Frey filament test, (b) paw withdrawal latency in cold allodynia test, (c) withdrawal latency in tail immersion test, (d) mechanical threshold in Randall–Selitto test, (e) withdrawal latency in hot plate test, (f) Rat Grimace scale and (g) immobility time in forced swim test. Each bar with a vertical line represents the mean ± S.D. of rats (n = 10) per group using one-way ANOVA followed by Tukey's post-hoc test (*p* < 0.05). CTRL; control, RIFM; reserpine-induced fibromyalgia model, SURM; suramin

Reserpine aggravated the allodynic response in rats. RIFM rats showed abnormalities in the pain response, noted by decreased the threshold of Von Frey filament, cold allodynia paw withdrawal latency, and tail withdrawal latency by 97.8% [F_(2,27)_ = 28.27*, p* < *0.0001*, η^2^ = 0.78], 51.2% [F_(2,27)_ = 27.02, *p* < *0.0001*, η^2^ = 0.64], and 74% [F_(2,27)_ = 86.33, *p* = 0.0001, η^2^ = 0.88], respectively, compared to the control rats (Fig. 1a-c). At *p* < 0.0001, intraperitoneal injection of SURM increased the withdrawal threshold of Von Frey filament to (34-fold), cold allodynia paw withdrawal latency to (1.6-fold) and tail withdrawal latency to (2.6-fold) compared with RES-treated animals, which depicted an anti-allodynic effect. Notably, SURM demonstrated a significant increase in pain threshold on day 7, with improvements observed in the Von Frey filament, cold allodynia paw, and tail withdrawal tests by 28-fold, 1.6-fold, and 2.4-fold, respectively, compared to pre-administration values of SURM. While the values of control and RES groups at days 3 and 7 did not signficantly changed. In Randall-Sellito and hot plate tests, RIFM rats significantly decreased the mechanical threshold of Randall-Sellito and withdrawal latency from the hot plate test by 72% [F_(2,27)_ = 77, *p* < *0.001*, η^2^ = 0.89] and 74.5% [F_(2,27)_ = 90.7*, p* = 0.0001, η^2^ = 0.9], respectively, relative to the control group (Fig. 1d-e). The anti-hyperalgesic effect of SURM reversed these changes to 2.8-fold and 2.3-fold, respectively. Additionally, a substantial increase in mechanical threshold was observed, with treated rats showing a 3.6-fold enhancement in both the Randall-Sellito and hot plate tests compared to baseline SURM values. Exacerbated spontaneous pain was evaluated by rat Grimace scale. There were significant increase in the four action units: changes in the features of the ears (5.3-fold) F_(2,27)_ = 37.31, *p* = 0.0001, η2 = 0.73; whiskers (1.5-fold) F_(2,27)_ = 35.53, *p* = 0.0001, η2 = 0.732; nose/cheek flattening (7.5-fold) F_(2,27)_ = 21.08, *p* < 0.0001, η2 = 0.6; and orbital tightening (8.5 fold) F_(2,27)_ = 43.53, *p* < 0.0001, η2 = 0.76, in comparison to the healthy rats. When SURM was administered, ear alterations were reduced by 73.6%, whisker changes dropped by 78%, nose/cheek flatness decreased by 52%, and orbital tightening lessened by 68% compared to RES-treated rats. (Fig. 1f). At *p* < 0.0001, RIFM rats presented depressive-like behavior revealed by prolonged immobility time in the forced swim test to reach 5.2-fold [F_(2,27)_ = 48.85, η^2^ = 0.78] compared to normal rats. SURM-treated rats increased mobility to 77% relative to the untreated rats (Fig. 1g).

### Suramin Restored Motor Coordination in the FM-Like Model

**Fig. 2:**
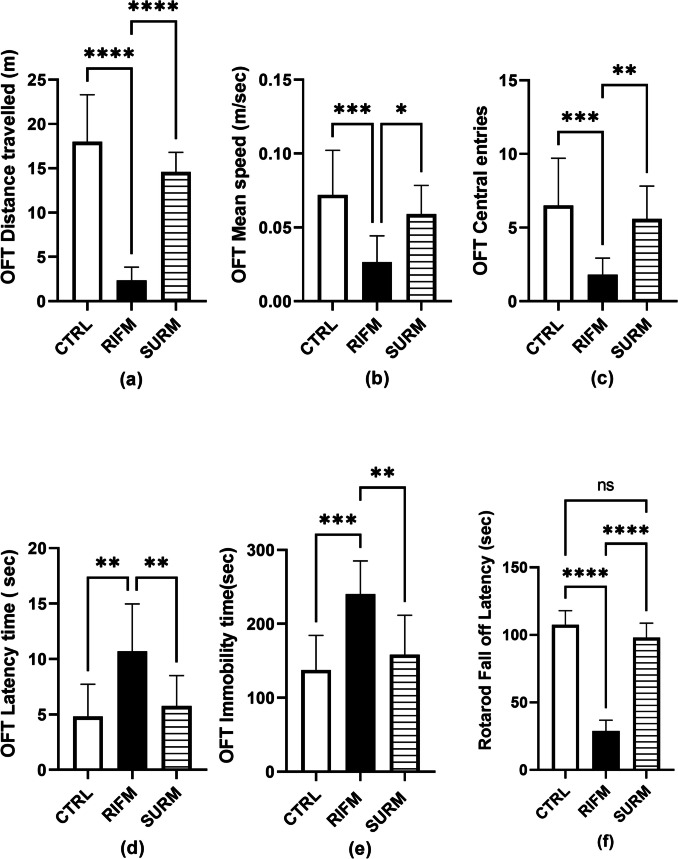
Effect of SURM on locomotor activity and motor coordination of RIFM rats in OFT and Rotarod test. Panels represent: (a) OFT distance traveled, (b) OFT mean speed, (c) OFT center zone entries number, (d) OFT latency time, (e) OFT immobility time and (f) rotarod falling off latency. Each bar with vertical line represents mean ± S.D. of rats (n = 10) per group, using one-way ANOVA followed by Tukey’s post hoc test; *p* < 0.05. OFT; open field test distance, CTRL; control, RIFM; reserpine-induced fibromyalgia model, SURM; suramin

Reserpine administered subcutaneously to rats resulted in motor alterations observed in the open field and rotarod tests (Fig. 2). Regarding the open field test, FM rats showed a significant decrease in OFT distance travelled by 82% F_(2,27)_ = 57.76, *p* = 0.0005, η^2^ = 0.81; OFT mean speed by 64.6% [F_(2,27)_ = 10.41, *p* = 0.0007, η^2^ = 0.43 and OFT central entries number by 72% F_(2,27)_ = 11.32, *p* = 0.0004, η^2^ = 0.45. These changes were accompanied by a significant increase in OFT latency time by 1.2-fold [F_(2,27)_ = 8.764, *p* = 0.0016, η^2^ = 0.39] and OFT immobility time by 74% [F_(2,27)_ = 12.48, *p* < 0.0002, η^2^ = 0.48] compared to healthy animals. However, SURM rats diminished motor alterations exhibited by the open field test to reach fivefold for OFT distance traveled (*p* = 0.0065); 1.2-fold for OFT mean speed (*p* = 0.0221); twofold for OFT central entries number (*p* = 0.0033); 45% for OFT latency time (*p* = 0.0016), and 34% for OFT immobility time (*p* < 0.0002). In the rotarod test, RIFM animals greatly decreased the rats'residence time on the rotarod by 73% [F_(2, 27)_ = 192.7, *p* < 0.0001, η^2^ = 0.93] compared to the healthy rats. SURM rats enhanced falling latency in RIFM to 2.4-fold (*p* < 0.0001).

### Suramin Alleviated Histopathological Changes in the Thalamus of FM-Like Model

**Fig. 3:**
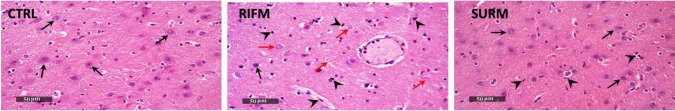
Effect of SURM on histopathological changes in RIFM rats. Representative photomicrographs illustrating H&E staining of the thalamus. Black arrows denote intact well-organized neurons with intact cytoplasmic and nuclear details, red arrows showed marked higher records of neuronal loss and necrotic structure less neurons while arrowhead showed significantly higher records of reactive glial cells dominated by microglial cell infiltrates. CTRL; control, RIFM; reserpine-induced fibromyalgia, SURM; suramin. Scale bar: 50 μM

Normal control samples demonstrated apparent intact well-organized neurons with intact cytoplasmic and nuclear details (black arrow), intact intercellular brain matrix with intact vasculatures and minimal abnormal glial cells infiltrates). On contrary, RIFM samples showed marked higher records of neuronal loss and necrotic structure less neurons (red arrow) alternated with fewer figures of apparent intact neurons (black arrow) and moderate vacuolization of intercellular brain matrix. Moreover, significantly higher records of reactive glial cells dominated by microglial cell infiltrates (arrowhead) (Fig. 3). However, SURM-treated rats showed significant neuroprotective efficacy with abundant figures of apparent intact neurons (black arrow) resembling normal control samples accompanied by minimal records of abnormal glial cells infiltrates (arrowhead) (Fig. 3).

### Suramin Counteracted the Elevated Purinergic Receptors in the Thalamus of the FM-Like Model

**Fig. 4:**
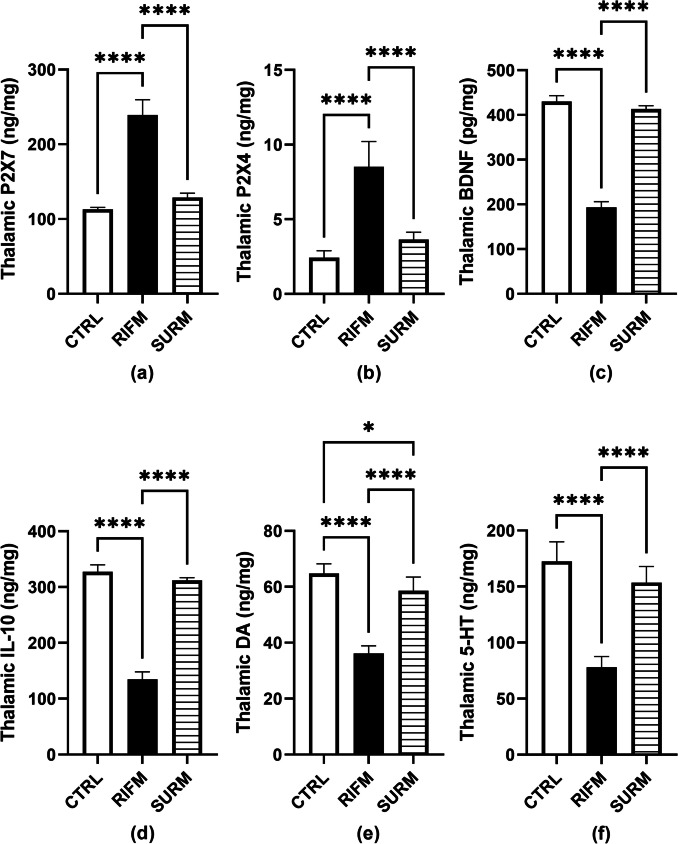
Effect of SURM on expression of Purinergic receptors, neurotrophic factor, anti-inflammatory cytokine and neurotransmitters levels in the thalamus of RIFM rats. Panels represent: (a) P2X7, (b) P2X4, (c) BDNF, (d) IL- 10, (e) DA and (f) 5-HT. Each bar with vertical line represents mean ± S.D. (*n* = 6), using one-way ANOVA followed by Tukey’s post hoc test; *p* < 0.05. CTRL; control, vs RIFM; reserpine-induced fibromyalgia model, vs SURM; suramin

RIFM animals exhibited overexpression of purinergic receptors in the thalamus. As shown in Fig. 4 a and 4b, P2X7 and P2X4 were overexpressed by 1.4-fold and 2.5-fold respectively, for P2X7 F_(2,15)_ = 187.9, *p* < 0.0001, η2 = 0.96; for P2X4 F_(2,15)_ = 56.65, *p* < 0.0001, η2 = 0.88, relative to the normal animals. On the other hand, SURM injection repressed P2X7 expression by 46% (*p* < 0.0001) and P2X4 expression by 57% (*p* < 0.0001) compared to the untreated group.

### Suramin Up-Regulated The Neurotrophic Factor and Anti-Inflammatory Cytokine in the Thalamus of the FM-Like Model

In Fig. 4c and 4 d, RIFM rats downregulated both the neurotrophic factor BDNF and the anti-inflammatory cytokine IL- 10 by 55% [F_(2,15)_ = 768.7, *p* < 0.0001, η^2^ = 0.99] and 58.7% [F_(2,15)_ = 590.1, *p* < 0.0001, η^2^ = 0.98], respectively, compared to the normal group. SURM-treated rats significantly countered this decrease in BDNF to (1.1-fold) and IL- 10 to (1.3-fold) compared to untreated animals (*p* < 0.0001).

### Suramin Affected Neurotransmitter Perturbations in the Thalamus in the FM-Like Model

As depicted in Fig. 4e and 4f, RES-treated animals exhibited a reduction in the monoamines DA and 5-HT by 44% and 54.8%, respectively (*p* < 0.0001). SURM-treated rats showed a reversal effect of this inhibition for DA and 5-HT to 61% [F_(2,15)_ = 93.19, *p* < 0.0001, η^2^ = 0.92] and 97% [F_(2,15)_ = 76.14, *p* < 0.0001, η^2^ = 0.91], respectively.

### Suramin Attenuated NLRP3 Inflammasome Complex and Pyroptotic Parameters in the FM-Like Model

**Fig. 5:**
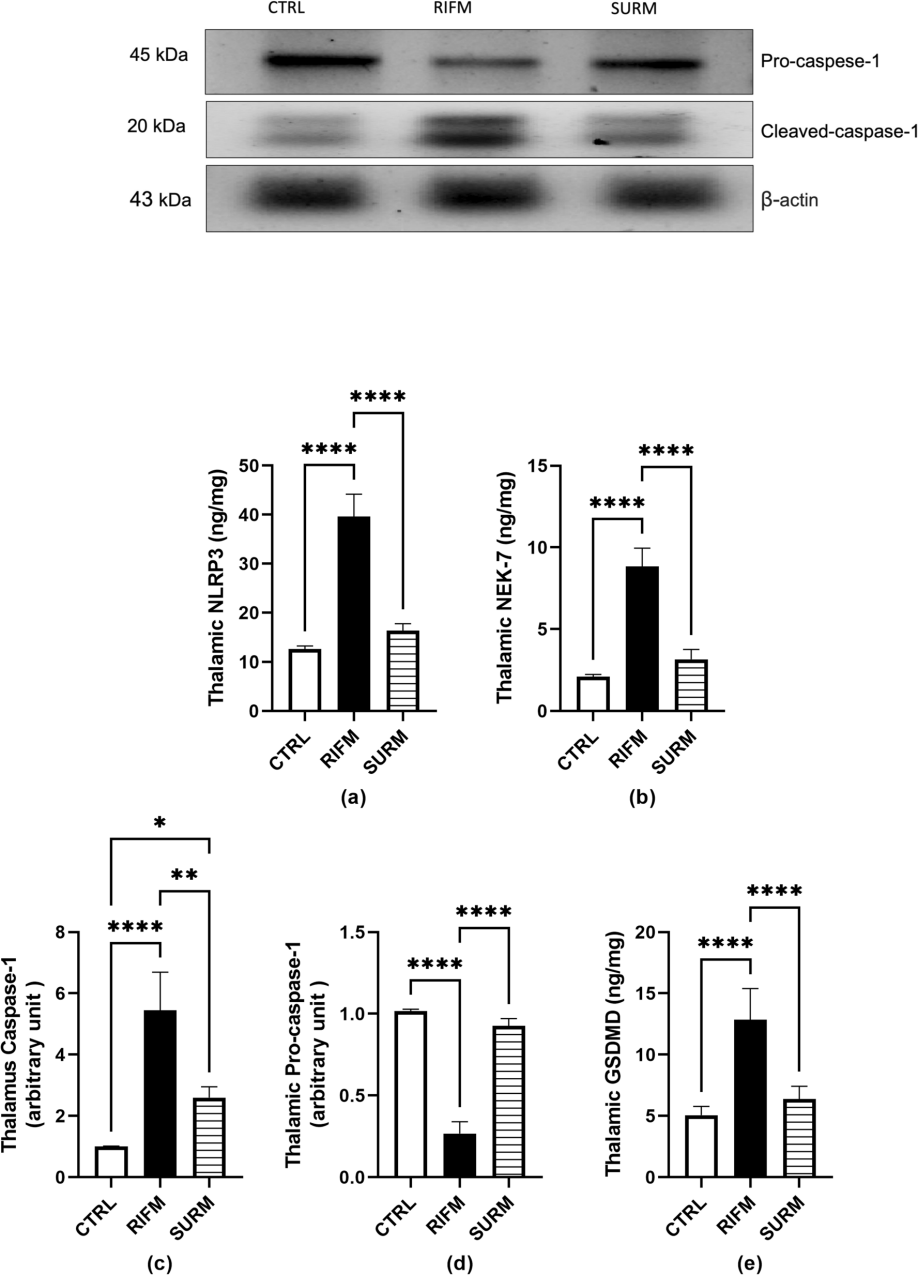
Effect of SURM on NLRP3 inflammasome complex and pyroptotic protein levels in RIFM rats. Panels represent: (a) NLRP3, (b) NEK- 7, (c) western blot and respective quantification of caspase- 1, (d) western blot and respective quantification of pro-caspase- 1, and (e) GSDMD. Each bar with vertical line represents mean ± S.D. of rats (*n* = 4–6), using one-way ANOVA followed by Tukey’s post hoc test; *p* < 0.05. CTRL; control, RIFM; reserpine-induced fibromyalgia model, SURM; suramin

**Fig. 6:**
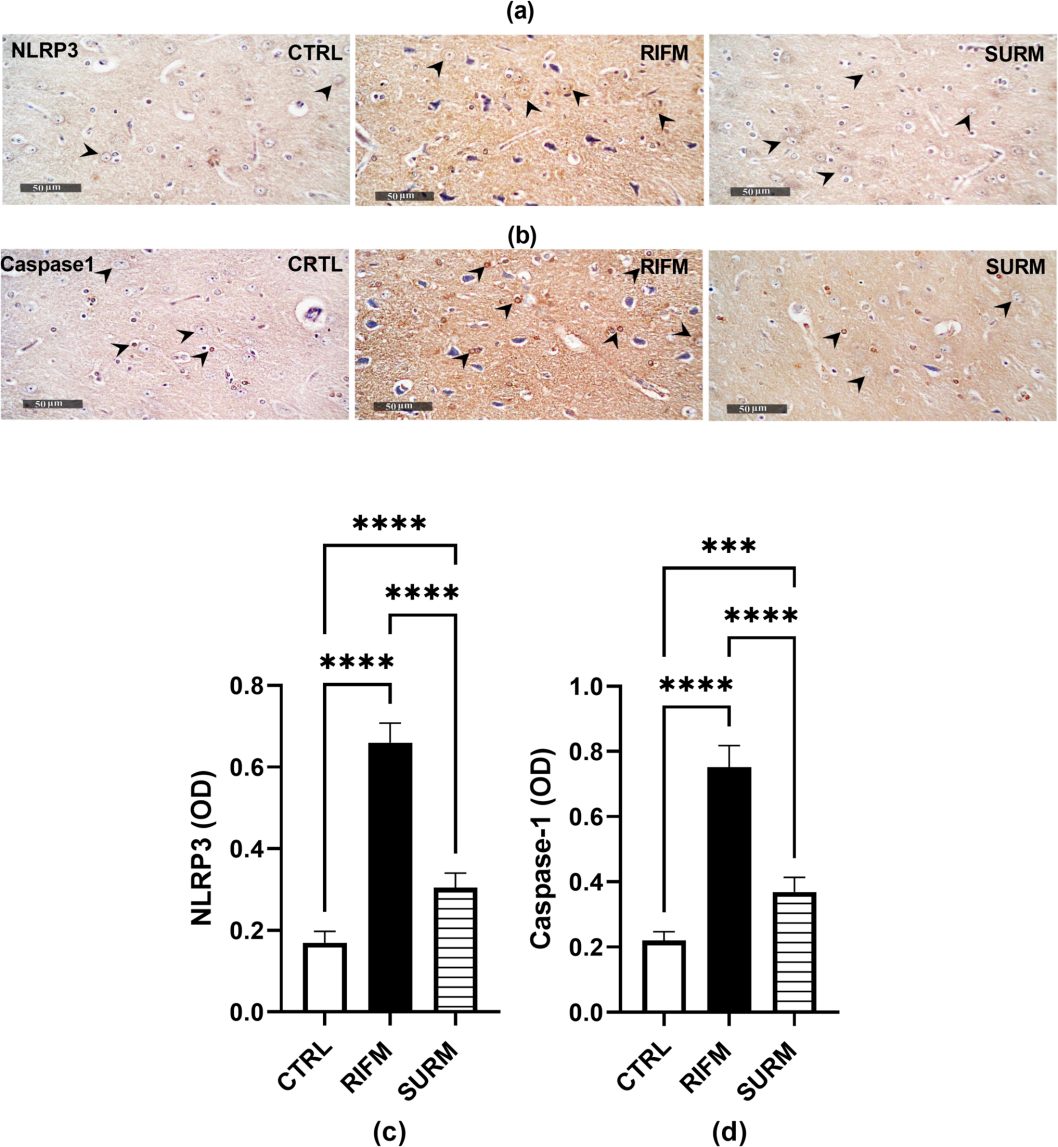
Effect of SURM on NLRP3 and Caspase- 1 immunohistochemical expressions in thalamus of RIFM rats. Immunohistochemical panels and their corresponding optical densities were represented as: (a,c) NLRP3, (b,d) caspase- 1, Each bar with vertical line represents mean ± S.D. of rats (n = 4), using one-way ANOVA followed by Tukey’s post hoc test; *p* < 0.05. CTRL; control, RIFM; reserpine-induced fibromyalgia model, SURM; suramin, NLRP3; nod-like receptor protein- 3

In RIFM rats, the NLRP3 inflammasome complex, including NLRP3, NEK7, and caspase- 1, was upregulated by twofold, 3.2-fold, and fourfold respectively, while pro-caspase- 1 expression decreased by 73.6% compared to normal rats [for NLRP3 F_(2,15)_ = 166.9, *p* < 0.0001, η^2^ = 0.95; for NEK7 F_(2,15)_ = 149, *p* < 0.0001, η^2^ = 0.95; for caspase- 1 F_(2,9)_ = 36.62, *p* < 0.0001, η^2^ = 0.89 and for pro-caspase- 1 [F_(2,9)_ = 274.1, *p* < 0.0001, η^2^ = 0.98] (Fig. 5a-d). Conversely, treatment with SURM downregulated these parameters NLRP3 (58.7%), NEK7 (64.2%) and caspase- 1 (52%) respectively, while upregulating pro-caspase- 1 to 2.4-fold. This was further confirmed by immunohistological examination of NLRP3 and caspase- 1, which showed upregulation by 2.8-fold and 2.4-fold respectively, in RIFM rats compared to healthy rats. In contrast, rats treated with Suramin showed downregulation of both NLRP3 and caspase- 1 by 53.7% and 51% [F_(2,15)_ = 267.5, *p* < 0.0001, η^2^ = 0.97; F_(2,15)_ = 188.3, *p* < 0.0001, η^2^ = 0.92], respectively, compared to untreated rats (Fig. 6). Due to NLRP3 activation, GSDMD is released, leading to GSDMD-mediated pyroptosis, a pro-inflammatory type of cell death. In the current study, GSDMD levels were elevated in untreated rats by 1.5-fold [F_(2,15)_ = 38.36, *p* < 0.0001, η^2^ = 0.83], while SURM injection reduced the increase to 50% (*p* < 0.0001) compared to untreated rats (Fig. 5e).

### Suramin Repressed Pro-Inflammatory Cytokines in the FM-Like Model

**Fig. 7:**
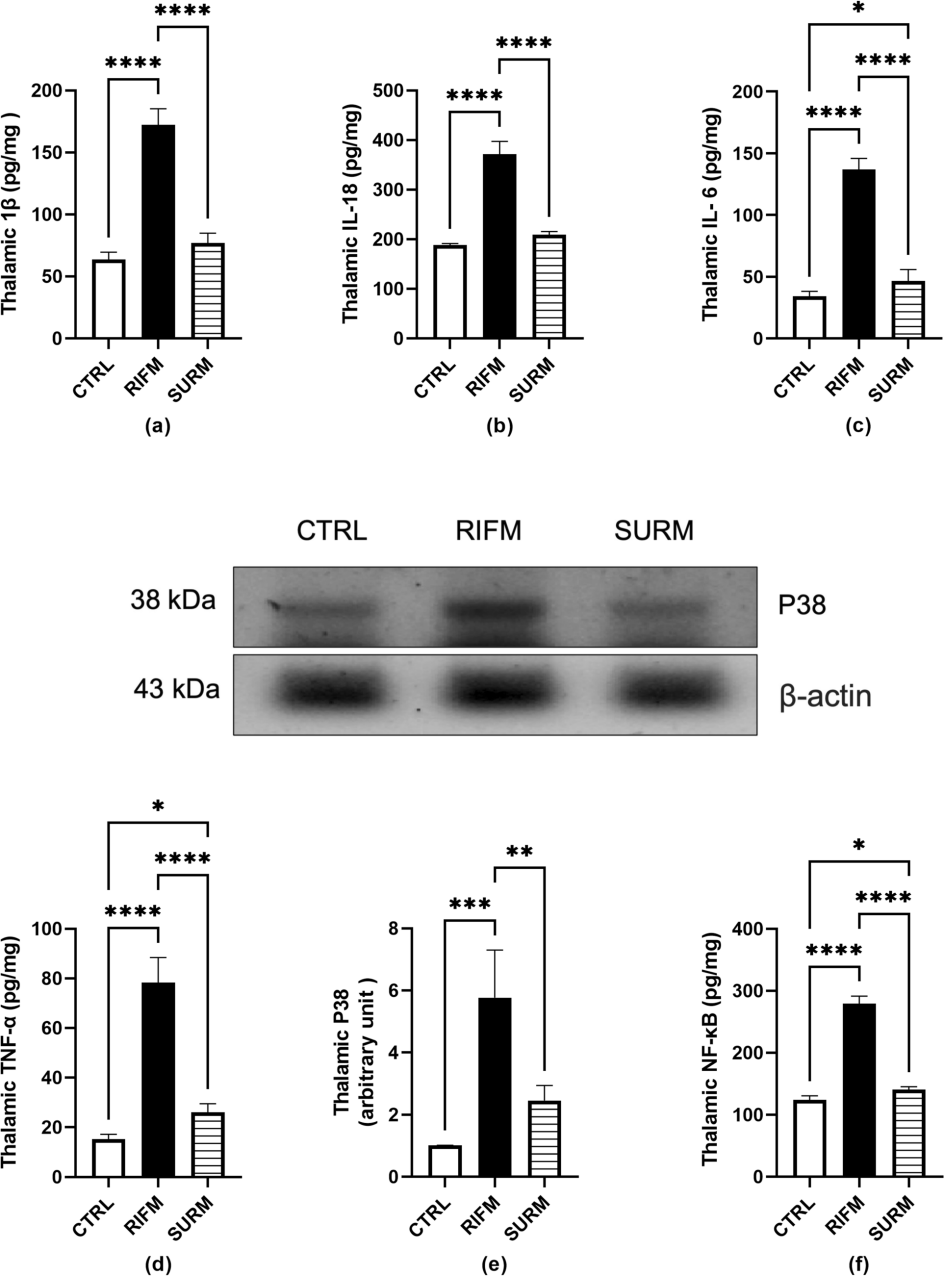
Panels represent: (a) IL- 1β, (b) IL- 18, (c) IL- 6, (d) TNF-α, (e) western blot and respective quantification of P38 and (f) NF-κB. Each bar with vertical line represents mean ± S.D. (n = 4–6), using one-way ANOVA followed by Tukey’s post hoc test; *p* < 0.05. CTRL; control, vs RIFM; reserpine-induced fibromyalgia model, vs SURM; suramin

Pro-inflammatory interleukins promote neuropathic pain in FM. RIFM rats showed an elevated level of IL- 1β (1.7-fold), IL- 18 (97%), IL- 6 (2.9-fold), and TNF-α (fourfold), for Il- 1β F_(2,15)_ = 234, *p* < 0.0001, η2 = 0.96; for IL- 18 F_(2,15)_ = 252.2, *p* < 0.0001, η2 = 0.97; for IL- 6 F_(2,15)_ = 323.3, *p* < 0.0001, η2 = 0.97 and for TNF-α F_(2,15)_ = 175.5, *p* < 0.0001, η2 = 0.95, as compared to the normal rats. Notably, SURM-treated rats countered this elevation to 55%, 43%, 65%, and 66.7% for IL- 1β, IL- 18, IL- 6, and TNF-α, respectively, compared to untreated rats (*p* < 0.0001) (Fig. 7a-d).

### Suramin Suppressed MAPK/NF-κB Pathway in the Thalamus in the FM-Like Model

Figure 7e revealed that phosphorylation of MAPK protein significantly increased in RIFM rats by 4.7-fold [F_(2,9)_ = 27.71, *p* < 0.0001, η^2^ = 0.86] compared to the control group. In contrast, SURM-treated rats suppressed this rise in p38MAPK protein to 57.4% (*p* = 0.0018). As depicted in Fig. 7f, RIFM rats showed an elevated level of NF-κB by 1.2-fold [F_(2,15)_ = 593.3, *p* < 0.0001, η^2^ = 0.98] compared to normal rats. Treatment with SURM reversed this effect by 98% relative to untreated rats (*p* < 0.0001). Figure 7 Effect of SURM on pro-inflammatory cytokines and MAPK/NF-κB pathway in the thalamus of RIFM rats.

### Suramin Modulated M1/M2 Macrophage Polarization Imbalance in the Thalamus of FM-Like Model

**Fig. 8:**
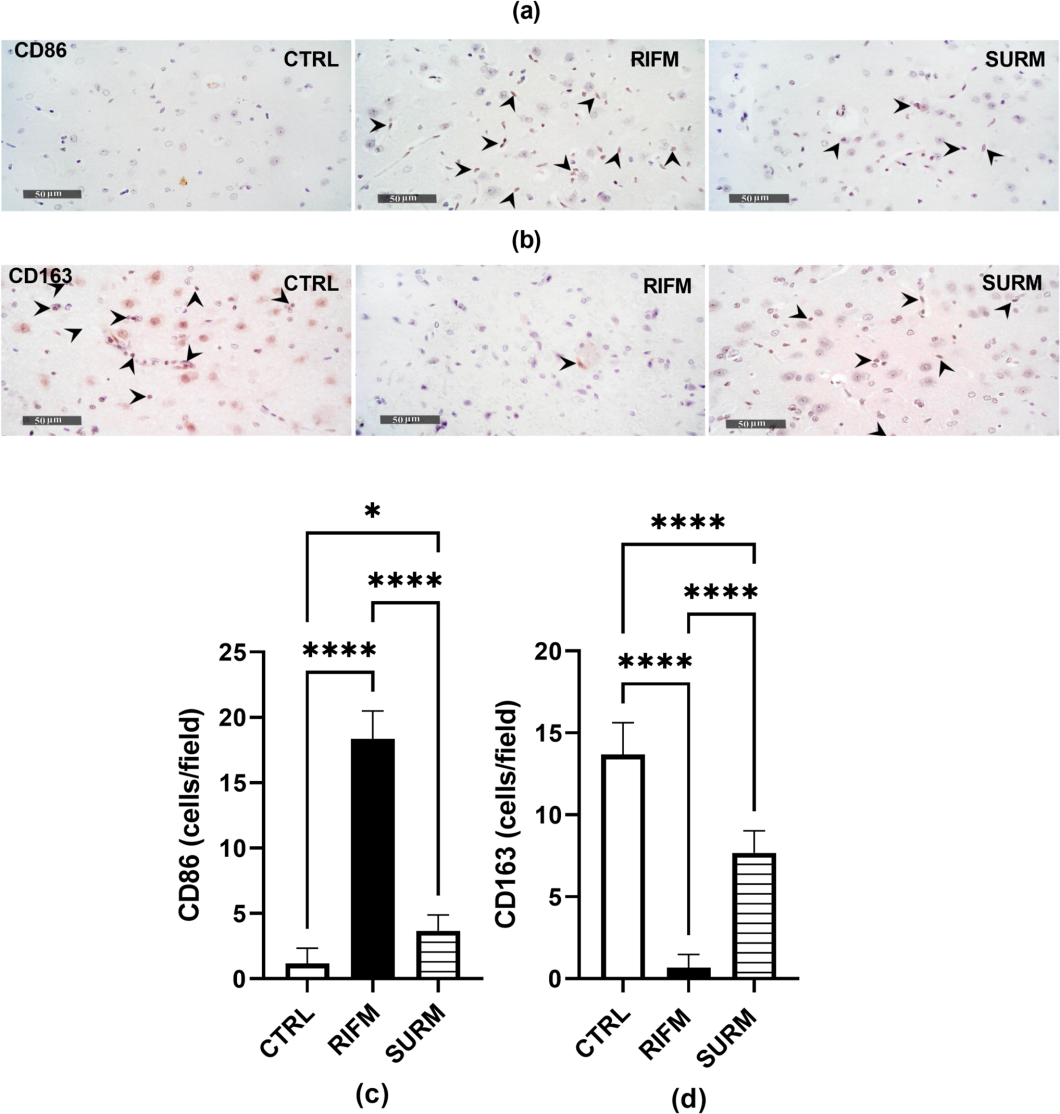
Effect of SURM on M1-M2 microglia polarization in the thalamus of RIFM rats. Immunohistochemical expressions and their corresponding optical densities were represented: (a,c) CD86 as M1 microglia marker, (b,d) CD163 as M2 microglia marker. Each bar with vertical line represents mean ± S.D. (*n* = 4), using one-way ANOVA followed by Tukey’s post hoc test; *p* < 0.05. CTRL; control, vs RIFM; reserpine-induced fibromyalgia model, vs SURM; suramin

Immunohistological analysis was utilized to assess the expressions of CD86 (M1 microglia marker) and CD163 (M2 microglia biomarker). In the thalamus, RES administration increased the expression of CD86 by 14.7-fold [F_(2,15)_ = 206.4, *p* < 0.0001, η^2^ = 0.96] compared to normal rats, while the thalamic level of CD163 decreased by 95%. This indicated a polarization of M1/M2 microglia towards the M1 phenotype. Surprisingly, treatment with SURM upregulated CD163 by 10.5-fold [F_(2,15)_ = 119.1, *p* < 0.0001, η^2^ = 0.94] compared to RIFM rats. Moreover, SURM exhibited significant anti-inflammatory activity by shifting the M1/M2 macrophage balance towards the M2 phenotype, as evidenced by the substantial downregulation of CD86 by 79.9% as shown in Fig. 8.

## Discussion

Fibromyalgia syndrome represents a severe manifestation of chronic, widespread pain. A clinical trial demonstrated alterations in thalamic interaction with the cortex, rendering FM brains more sensitive to external stimuli and amplifying pain perception (Kim et al. 2021). Flodin et al. (2014) identified a correlation between elevated connectivity between the left thalamus and prefrontal cortex and heightened pain sensitivity, indicative of hyperalgesia (Flodin et al. 2014). Notably, the data in the present study align with prior research. Histopathological examination of the brains of RES-treated rats using hematoxylin and eosin staining revealed morphological changes in the thalamus compared to healthy rats. Furthermore, this study demonstrated changes in nociception induced by RES injection, evidenced by decreased mechanical allodynia threshold in the Von Frey filament test, cold allodynia paw withdrawal latency, and tail withdrawal latency in the tail immersion test. These findings indicate a pronounced allodynia response. Simultaneously, the reduced Randall-Sellito mechanical threshold and hot plate withdrawal latency confirmed a significant hyperalgesia response. All these evoked pain responses were in concordance with heightened spontaneous pain as observed by the rat Grimace scale in the present study. This scale focuses on specific facial expressions, which are crucial for ensuring the translational relevance of preclinical study findings. The study acknowledges that the RIFM model does not fully replicate the chronic nature of pain or encompass the broad spectrum of factors contributing to FM in humans. Specifically, it may not capture long-term immune responses, genetic predispositions, or the impact of early-life trauma often seen in FM patients. The present study focused on assessing pain at its peak on day 7 to evaluate the efficacy of SURM in alleviating nociceptive symptoms. However, the efficacy of SURM using four and six reserpine injection protocols should be evaluated in future studies to explore its effects on prolonged pain, and to elucidate the full therapeutic potential of P2X inhibition (Nagakura et al. 2012).

P2X receptors are widely distributed throughout the human body and function as ATP-gated cation channels. These receptors are classified into seven subtypes (1–7), with P2X7 and P2X4 closely associated with the onset of various diseases (Di Virgilio et al. 2017; Stokes et al. 2017). P2X7 is prominently expressed in microglia, playing a significant role in driving neuroinflammation (Adinolfi et al. 2018). Similarly, P2X4 receptors are abundantly found in neurons and glial cells in several brain regions, including the thalamus (Montilla et al. 2020). Studies have shown that P2X7 and P2X4 receptors are closely linked to pain mechanisms (Ulmann et al. 2010; Hu et al. 2022). Interestingly, in the current study, inhibition of P2X7 and P2X4 receptors by SURM alleviated hyperalgesia in the Randall-Selitto and hot plate tests, as well as allodynia in the Von Frey filament, cold allodynia, and tail immersion tests. This demonstrated significant anti-hyperalgesic and anti-allodynic responses to purinergic receptor inhibition, highlighting the pivotal role of purinergic receptors in pain transmission through the thalamic tract in fibromyalgia. The co-expression of P2X4 and P2X7 receptors enhances the P2X7 receptor-mediated inflammatory response and facilitates P2X7 receptor-mediated IL- 1β production and inflammation through calcium influx (Kawano et al. 2012). Activation of P2X7 is critical in the assembly of the NLRP3 inflammasome and the subsequent release of pro-inflammatory cytokines like IL- 1β and IL- 18 (Di Virgilio et al. 2017). Importantly, this process requires calcium influx triggered by the P2X4 receptor (Sakaki et al. 2013), illustrating the neuroprotective effect of purinergic receptor blockade against the release of pro-inflammatory cytokines. SURM’s downregulation of P2X receptors may result from a pleiotropic effect involving genetic modulation, inflammation resolution, apoptosis inhibition, and receptor internalization. SURM has been shown to destabilize mRNA, reducing the expression of proteins like IL- 18 and inhibiting RNA polymerase activity, suggesting an indirect influence on P2X receptor (Kakuguchi et al. 2018; Wiedemar et al. 2020; Oda et al. 2023). In inflammatory environments, cellular ATP release enhances purinergic signalling that needs to impede this vicious cycle. However, by lowering pro-inflammatory cytokines (e.g., IL- 6, IL- 1β) and promoting a shift to an anti-inflammatory macrophage phenotype, SURM creates conditions less dependent on P2X receptor expression (Gu et al. 2000; Liu et al. 2012). The P2X7 gene is upregulated in response to inflammatory signals like interferon-gamma (IFN-γ) (Gu et al. 2000) which drives M1 macrophage polarization. SURM has shown anti-inflammatory action through reducing IFN-γ, facilitating a shift toward the M2 phenotype (Novales-Li 1996). This suggests that the downregulation of P2X receptors is likely due to a reduced inflammatory state, rather than direct inhibition by SURM. Moreover, its anti-apoptotic effects further reduce ATP-driven purinergic signaling, minimizing receptor upregulation (Eichhorst et al. 2004; Liu et al. 2021). Lastly, SURM’s competitive inhibition induces P2X receptor internalization, reducing receptor density on the cell surface (Bobanovic et al. 2002). These mechanisms may illustrate SURM's complex role in modulating P2X receptor presence and activity.

Previous research has indicated the critical role of the NLRP3 inflammasome in pain plasticity and its involvement in inflammatory disorders. The NLRP3 inflammasome is a crucial signaling hub regulating the production of IL- 1β and IL- 18, two pro-inflammatory cytokines of the IL- 1 family (Jo et al. 2016). Inflammasome activation is regulated by two signals: the Toll-like receptor (TLR)/nuclear factor (NF)-κB pathway as the first signal, and the second signal involving damage-associated molecular patterns (DAMPs) and pathogen-associated molecular patterns (PAMPs). These signals converge to form a multi-protein complex comprising pro-caspase- 1, NLRP3, and the adaptor protein ASC, activating the functional NLRP3 inflammasome (Hornung and Latz. 2010). Upon assembly of the NLRP3 inflammasome, inactive caspase- 1 is cleaved into its active form by proteases. In the present study, ELISA and tissue-based immunohistochemical analyses demonstrated reduced immunoreactivity for both NLRP3 and active caspase- 1 in the thalamus of SURM-treated rats compared to untreated animals. This reduction may be attributed to inhibition of the TLR/NF-κB pathway or P2X7/P2X4 receptors. NEK7, a family member of the NIMA-related kinases (NRKs), regulates NLRP3 activity. SURM inhibits P2X7, which controls NEK7, potentially contributing to the reduced NLRP3 expression. Notably, the P2X4 receptor regulates NLRP3 protein production in a diabetic nephropathy model (Chen et al. 2013), and its activation can activate NF-κB; upstream of NLRP3 (Xing et al. 2023). NLRP3 gene expression may be upregulated in FM rats due to NF-κB or P2X4, whereas SURM administration blocks this effect (Shen et al. 2023*)*. The study acknowledges that the fluctuating levels of ovarian hormones in the estrous cycle may influence NLRP3 inflammasome activity and nociceptive mechanisms. The study design utilized female rats to model the high prevalence of FM in women, as it predominantly affects females, with a reported female-to-male ratio of 3:1 (Clauw 2009; Silverman et al. 2010; Briones-Vozmediano 2016). The study’s primary focus was to evaluate the therapeutic effects of SURM in a RIFM model rather than to assess gender-specific effects related to the estrous cycle. Moreover, prior research indicates that RES disrupts the estrous cycle, typically inducing metestrus/diestrus-like conditions characterized by lower estrogen levels (Barraclough and Sawyer 1959). Thus, after RES administration, all rats were likely subjected to similar endocrine conditions, minimizing variability associated with estrous cycle fluctuations (Krulich et al. 1975). Although estrous phases were not monitored, the study aimed to control for hormonal influence using group averages and validated sample sizes to evenly distribute any residual variability. This approach aligns with findings from Hernandez-Leon et al. (2018), which suggest that sex differences are not key determinants of FM-like pain behaviors in RES-treated rats (Hernandez-Leon et al. 2018).

Earlier research has investigated the NF-κB/NLRP3 inflammasome signaling pathway as a specific target for managing neuropathic pain (Zhang et al. 2017). This idea stems from previous findings indicating that NF-κB signaling plays a crucial role in nociception in chronic pain disorders like fibromyalgia (Kaur et al. 2019) and other chronic pain conditions (Luo et al. 2020). Immunohistochemical studies on fibromyalgia muscles have shown strong expression of NF-κB, with IL- 1β and IL- 18 being NF-κB-dependent pro-inflammatory cytokines (Cordero et al. 2014). Using the RIFM rat model, it becomes possible to demonstrate a positive correlation between NF-κB and NLRP3 inflammasome activation in promoting the production of inflammatory cytokines, leading to hyperalgesia. Interestingly, SURM-treated rats exhibited inhibition of NF-κB and subsequent inflammasome activation. This inflammatory cascade contributes to resting macrophage transition towards M1 activation over M2, leading to neuroinflammation. In neuropathic pain models, high expression of inflammatory M1 macrophage-specific molecules and low levels of M2 microglia-specific molecules indicate M1/M2 microglia polarization shift (Kiguchi et al. 2015; Atta et al. 2023a). In fibromyalgia patients, nociplastic pain arises due to M1 microglia-dependent neuroinflammation (Atta et al. 2023b). This aligns with the current study's findings, as immunohistological results in RIFM rats showed a shift towards the M1 phenotype, as seen in CD163, a marker of M1 microglia. Remarkably, after inhibiting NF-κB signaling with SURM, M1-polarized microglia decreased, and M2-polarized microglia increased in the thalamus of RIFM rats. This reversal in microglia polarization aligns with the highly dynamic nature of this process observed in both healthy and pathological conditions. Additionally, this aligns with Suramin's in vitro anti-inflammatory benefits in intervertebral disc degeneration, partly linked to its interference with NF-κB signaling (Liu et al. 2021).

Caspase- 1 is critical in pyroptosis, an inflammatory form of cell death. Pyroptosis involves gasdermin D (GSDMD) and interleukins such as IL- 1β and IL- 18. Active caspase- 1 converts pro-IL- 1β and pro-IL- 18 into their biologically active forms. Additionally, active caspase- 1 cleaves GSDMD, creating pores in the plasma membrane that enable the release of IL- 1β and IL- 18. In the current study, RIFM rats exhibited significantly elevated levels of GSDMD compared to normal animals. This increase may be attributed to P2X7 and P2X4 receptors. This aligns with previous findings showing that silencing P2X7 alleviates pyroptosis-associated conditions like abdominal aortic aneurysm (Sun et al. 2023). Mechanistically, silencing P2X4 blocks the NLRP3/caspase- 1 pathway, demonstrating a neuroprotective effect in an intracerebral hemorrhage mouse model, while administration of an NLRP3 activator induces neuronal impairment in the same model (Wu et al. 2023). This indicates a strong relationship between P2X4 and the NLRP3 inflammasome. Furthermore, activation of the P2X7 receptor leads to MAP kinase (MAPK) activation downstream (Suzuki et al. 2004). Studies on thalamic injury (Deng et al. 2023) have highlighted the involvement of microglial p38-MAPK in neuropathic pain development. SURM-treated rats showed a significant reduction in p38 expression compared to RIFM rats. This may explain SURM's behavioral effects, as neuropathic pain and central sensitization are mediated by the activation of P2X7/P2X4 receptors and p38-MAPK (Ji and Suter 2007).

IL- 1β and IL- 18 are involved in neuroinflammation and pain processes peripherally and centrally. Notably, serum samples from FM patients have shown elevated levels of IL- 1β and IL- 18. Furthermore, IL- 1β serum levels correlate positively with FM patients'pain scale scores (Cordero et al. 2014). The current study aligns with these previous findings, as SURM administration reduced the expression of IL- 1β and IL- 18 in the thalamus compared to RIFM rats. This aligns with research showing that inhibiting the thalamic NLRP3 inflammasome reduces IL‐18 and IL‑1β, leading to behavioral alleviation of thermal and mechanical pain sensitivity in a central post-stroke pain mouse model (Huang et al. 2022). The pro-inflammatory cytokine IL- 1β may affect BDNF expression through P2X4 receptor activation on microglia (Guo et al. 2020). This may attenuate BDNF's neuroprotective effects by IL- 1β-mediated MAPK activation, as observed in in vitro studies (Tong et al. 2008). In the current study, RIFM rats exhibited decreased BDNF expression, while SURM-treated rats showed a reversal of this decrease, indicating a potential neuroprotective effect of SURM. Typically, P2X4 receptor is activated in pain disorders that followed by the release of both BDNF and IL- 1β from microglia. This drives neuroinflammatory responses and enhances pain transmission. However, in the present study, P2X4 blocking by SURM promoted an M1-to-M2 microglial shift, associated with increased BDNF and reduced IL- 1β levels, suggesting an anti-inflammatory effect contrary to typical P2X4-mediated BDNF release. This may be due to the investigated brain area where supraspinal BDNF can exert neuroprotective and anti-inflammatory effects opposite to the pro-nocipetive character of BDNF observed in spinal regions. Thus, the observed inhibition of IL- 1β may allow BDNF’s beneficial actions to predominate. However, further assessment of P2X4 receptor behavior in microglia within the thalamus could provide additional insight into its role in enhancing fibromyalgia-related pain mechanisms via IL- 1β.

It is crucial to mention that BDNF is often linked to pain facilitation via neuronal excitation (Laske et al. 2007; Huang et al. 2021; Behnoush et al. 2023; Thakkar and Acevedo 2023), research also suggests it can play an anti-nociceptive and neuroprotective role, particularly within the CNS. Indeed, BDNF promotes neuronal survival and has been shown to increase serotonin synthesis in the brainstem, offering analgesic effects (Martin-Iverson et al. 1994; Yu et al. 2019). Additionally, other studies demonstrated that pain is often associated with reduced BDNF levels in supraspinal regions, such as the anterior cingulate cortex, which plays a critical role in pain perception (Al-Amin et al. 2011; Liu et al. 2024). This aligns with the present findings, suggesting that the observed upregulation of BDNF in the thalamus following SURM administration may contribute to its anti-nociceptive effects. Moreover, studies in other models of neuropathic pain have demonstrated BDNF's ability to reverse chronic pain signals in the spinal dorsal horn, besides emerging evidence points to its anti-inflammatory effects on microglia, further supporting its role in pain resolution (Cejas et al. 2000; Charlton et al. 2023). Interestingly, FM models have shown a reduction in BDNF levels following reserpine administration, as well as those using chronic restraint and intermittent cold stress (Lee et al. 2017, 2018; Roversi et al. 2019; Yu et al. 2019; Kang et al. 2024). In conclusion, while BDNF has pro-nociceptive effects by sensitizing primary afferent neurons and promoting pain hypersensitivity, substantial evidence supports its anti-nociceptive potential in CNS contexts. Future studies are warranted to investigate the long-term effects of SURM on BDNF dynamics to further understand its neuroprotective and pain-modulating properties.

Additionally, the delayed production of IL- 10 has been implicated in promoting hyperalgesia. Nerve injury often leads to an imbalance between pro-inflammatory cytokines such as IL- 1β, IL- 18, IL- 6, and TNF-α, and anti-inflammatory cytokines like IL- 10 (Alcami 2003). In FM rats, elevated levels of TNF-α and IL- 6 in the thalamus may contribute to hyperalgesia. This heightened inflammatory state could explain depressive behavior, similar to observations in post-mortem studies of depressed suicide victims (Suzuki et al. 2019) and preclinical studies (Zaky et al. 2024). Previous studies have linked elevated allodynia and hyperalgesia in FM rats to depressive-like behavior (Blasco-Serra et al. 2015). However, treatment with SURM reduced these cytokine levels, indicating an anti-inflammatory response through increased IL- 10 production. SURM-treated rats showed reduced immobility time in the forced swim test, suggesting an anti-depressive effect. This could be attributed to the depression of pro-inflammatory cytokines or P2X7 inhibition, as seen with the blockade of P2X7 receptors using brilliant blue G, which reduced depressive-like behavior in RIFM rats (D’Amico et al. 2021). Furthermore, treatment with SURM restored catecholamine levels in the thalamus of rats. This restoration may be linked to NLRP3 inhibition, as observed in studies where stem cell treatment restored brain monoamines in FM models (Mokhemer et al. 2023).

While the present study postulated that the thalamic mechanisms are beyond the SURM’s antinociceptive effects, it is important to consider that SURM may exert its influences through multiple sites and pathways. SURM's ability to inhibit P2X receptors suggests that it could modulate neuronal and glial interactions. Satellite glial cells (SGCs) are crucial for pain signaling and are integral to central sensitization. For instance, research has demonstrated upregulated P2X receptor expression in SGCs contributes to pain modulation (Wang et al. 2019; Hanani and Spray 2020). Moreover, the activation of P2X7Rs on SGCs extends to excite adjacent dorsal root ganglia (DRG) neurons which contribute to pain hypersensitivity in both acute and chronic pain. Thus, it is possible that SURM targeting to P2X receptor could affect SGCs-DRG neuronal interactions which was the rationale beyond peripheral sensitization reduction. Another possible mechanism is the targeting of spinal microglial P2X receptors by SURM, which may influence microglia-spinal dorsal horn neuron interactions. By suppressing microglial activation and the subsequent release of pro-inflammatory cytokines, SURM could be able to reduce central sensitization. These broader mechanisms may collectively contribute to the alleviation of FM symptoms observed in the present study. Future studies involving direct administration of SURM into thalamus or investigating SGC-DRG neuronal interaction or microglia-SDH neuron interaction after SURM administration would provide more definitive evidence of the site-specific actions of SURM.

## Conclusion

In summary, this study demonstrates the involvement of ATP induction via P2X7 and P2X4 receptor activation in reserpine-induced fibromyalgia (RIFM), leading to inflammatory responses that worsen FM symptoms. Additionally, the study provides initial evidence that SURM mitigates FM-associated pain, microglial-induced inflammation, and subsequent depressive episodes caused by RES. Future clinical trials may find this treatment approach appealing due to its anti-inflammatory effects, potentially through the inhibition of NLRP3 inflammasome signaling. This offers new insights into the potential role of SURM in managing fibromyalgia symptoms in patients.

## Supplementary Information

Below is the link to the electronic supplementary material.

Supplementary file1 (JPG 1192 KB)

Supplementary file2 (JPG 904 KB)

Supplementary file3 (JPG 830 KB)

## Data Availability

All relevant data are present in the submitted manuscript, table, and figures. Any other data will be made available on request.
