# Supplementary figures and images for: Possible Interaction of Suramin with Thalamic P2X Receptors and NLRP3 Inflammasome Activation Alleviates Reserpine-Induced Fibromyalgia-Like Symptoms
Source: J Neuroimmune Pharmacol. 2025 May 7;20(1):51. doi: 10.1007/s11481-025-10207-4 (PMC12055955; doi:10.1007/s11481-025-10207-4)

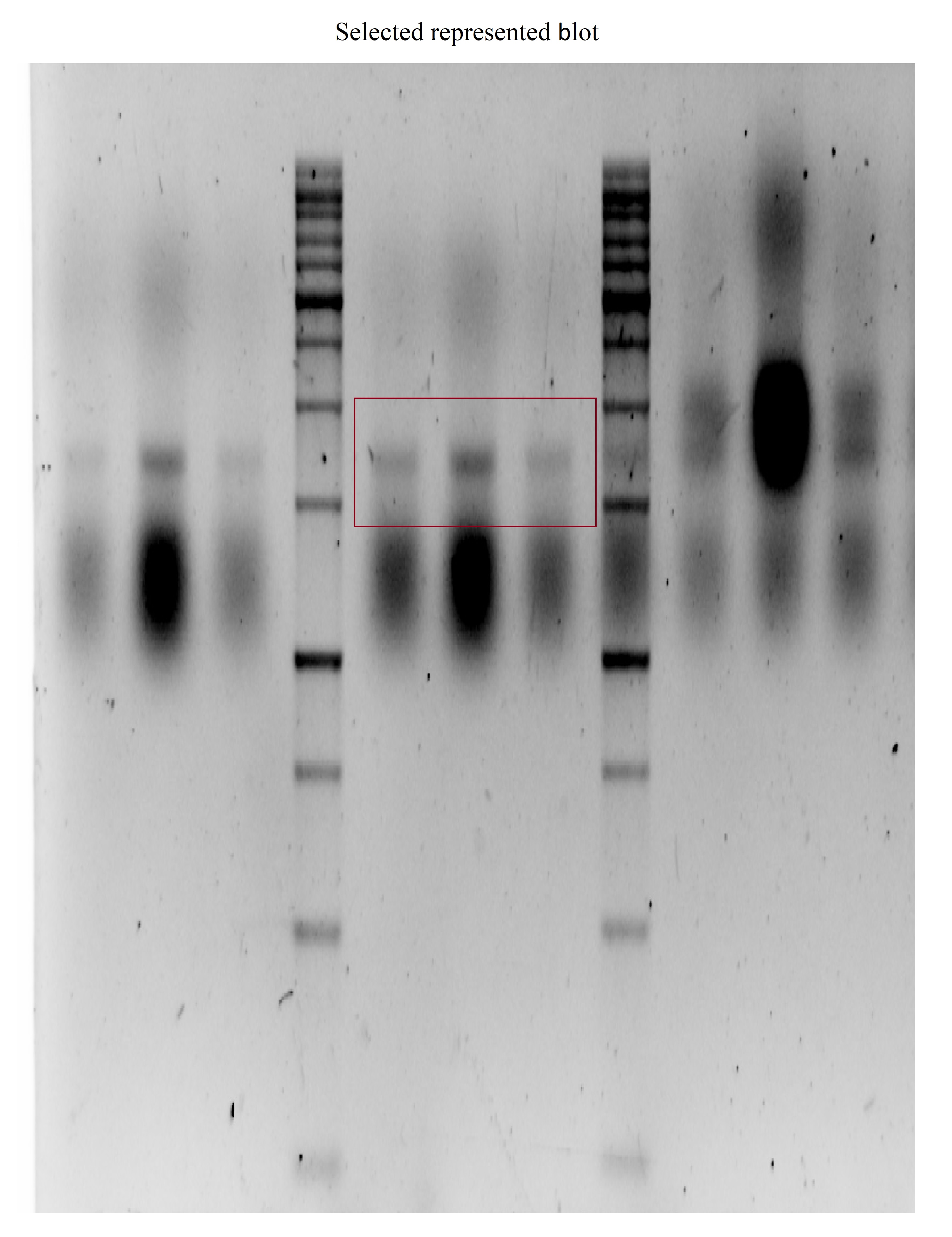

Supplement: Supplementary file 1 — Supplementary file1 (JPG 1192 KB) [file 11481_2025_10207_MOESM1_ESM.jpg]

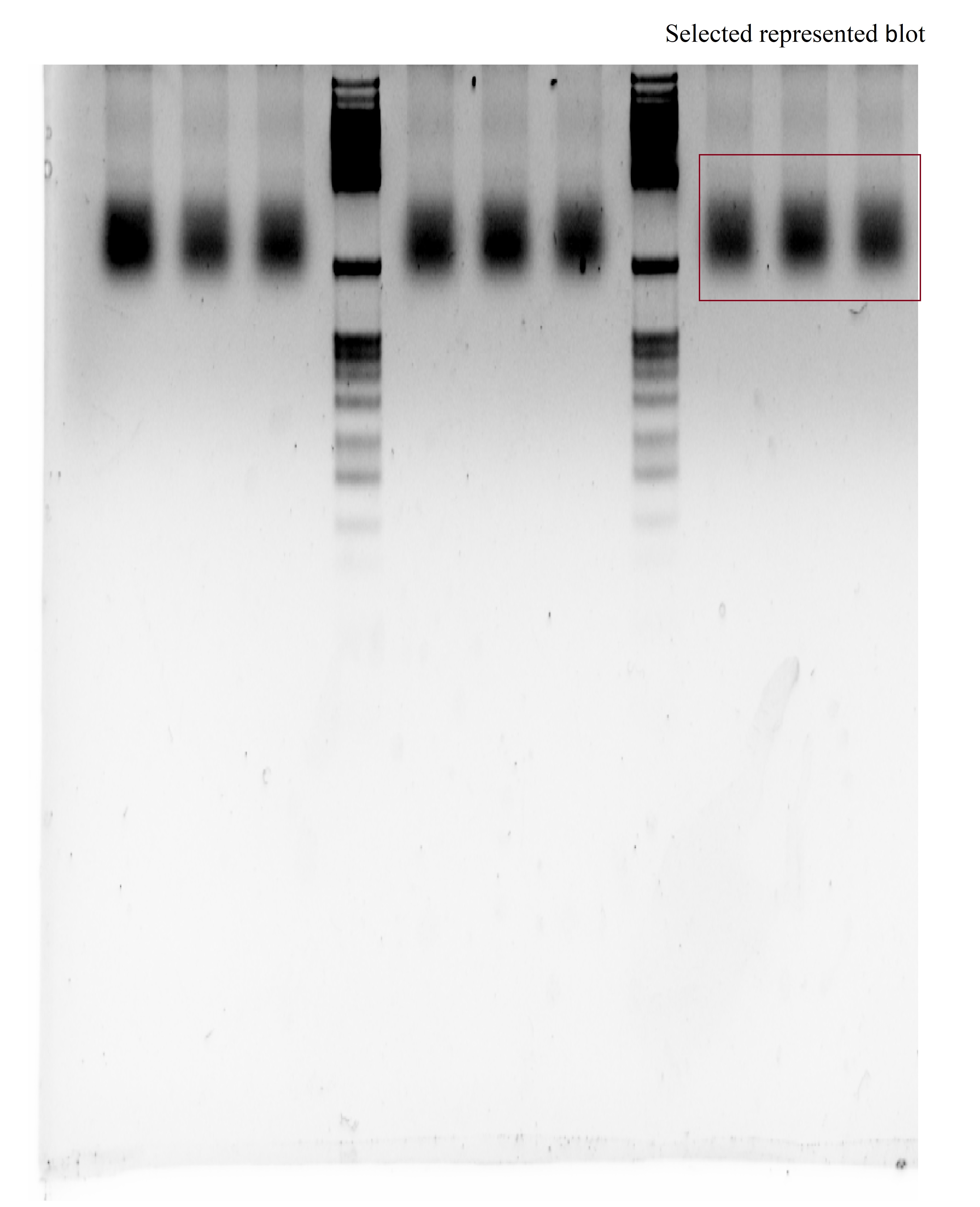

Supplement: Supplementary file 2 — Supplementary file2 (JPG 904 KB) [file 11481_2025_10207_MOESM2_ESM.jpg]

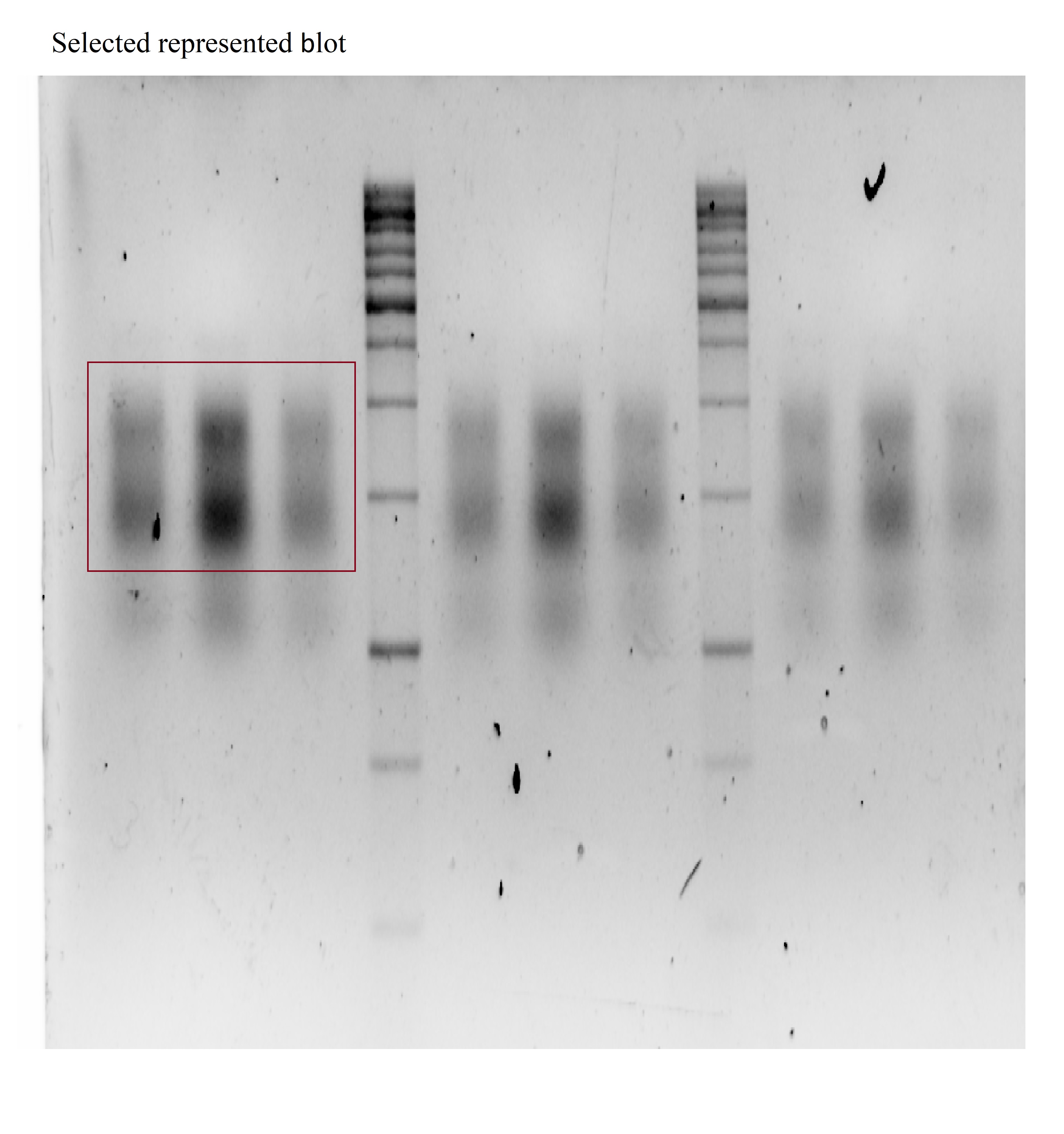

Supplement: Supplementary file 3 — Supplementary file3 (JPG 830 KB) [file 11481_2025_10207_MOESM3_ESM.jpg]
